# Prognostic accuracy of point-of-care ultrasound in patients with pulseless electrical activity: a systematic review and meta-analysis

**DOI:** 10.1186/s13049-025-01327-0

**Published:** 2025-02-10

**Authors:** Shang-Jun Zhang Jian, Tzu-Heng Cheng, Chieh-Ching Yen

**Affiliations:** 1 Department of Emergency Medicine, Chang Gung Memorial Hospital, Linkou Branch, Taoyuan, Taiwan; 2 Department of Emergency Medicine, New Taipei Municipal Tucheng Hospital, New Taipei City, Taiwan; 3 Institute of Emergency and Critical Care Medicine, National Yang Ming Chiao Tung University, Taipei, Taiwan

**Keywords:** Point-of-care ultrasound, Pulseless electrical activity, Cardiac arrest, Meta-analysis

## Abstract

**Background:**

The prognosis for pulseless electrical activity (PEA) is typically poor; however, patients with cardiac activity observed on point-of-care ultrasound (POCUS) tend to have better outcomes compared to those without. This systematic review and meta-analysis were conducted to assess the prognostic accuracy of cardiac activity detected by POCUS in predicting resuscitation outcomes in patients experiencing PEA.

**Methods:**

A comprehensive literature search was conducted in PubMed, Embase, and the Cochrane Central Register of Controlled Trials to identify relevant studies. The pooled sensitivity, specificity, positive likelihood ratio (PLR), negative likelihood ratio (NLR), diagnostic odds ratio, and the area under the summary receiver operating characteristic curve (SROC) were calculated using the bivariate model.

**Results:**

Eighteen studies comprising 1202 patients were included in the meta-analysis. Cardiac activity observed on POCUS demonstrated a pooled sensitivity of 0.86 (95% CI 0.67–0.95) and specificity of 0.64 (95% CI 0.51–0.75) for predicting return of spontaneous circulation, a pooled sensitivity of 0.89 (95% CI 0.80–0.94) and specificity of 0.73 (95% CI 0.63–0.81) for survival to admission (SHA), and a pooled sensitivity of 0.79 (95% CI 0.58–0.91) and specificity of 0.58 (95% CI 0.47–0.68) for survival to discharge. The highest area under the SROC, 0.89 (95% CI 0.86–0.92), was observed for SHA.

**Conclusions:**

Our study suggests that POCUS may serve as a vital component of a multimodal approach for early termination of resuscitation.

**Supplementary Information:**

The online version contains supplementary material available at 10.1186/s13049-025-01327-0.

## Introduction

Pulseless electrical activity (PEA), also known as electromechanical dissociation (EMD), is not uniformly defined but is often described as the presence of organized electrical activity on an electrocardiogram (ECG) monitor without a palpable pulse. The incidence of PEA arrest ranges from 19 to 23% among other types of cardiac arrest, with an increasing trend in recent years [1]. The rate of survival to hospital discharge for PEA arrest is around 8%, which is extremely low compared to 30.5% for shockable rhythms, warranting much more attention [1]. Pseudo-PEA was first described in 1992 by Paradis, N.A., et al., where, despite the absence of a palpable pulse, organized cardiac activity was observed during an echocardiogram [2]. It is believed that pseudo-PEA represents a state of profound shock, causing hemodynamic compromise and an inability to maintain perfusion pressure, leading to a nondetectable pulse. According to previous studies, pseudo-PEA has a better prognosis than true PEA [3–5]. Therefore, the rapid identification of pseudo-PEA is essential.

Point-of-care ultrasound (POCUS) has gained popularity in emergency departments (EDs) for diagnosing and guiding resuscitation in recent years due to its accessibility and non-invasiveness [6, 7]. The 2015 American Heart Association (AHA) Guidelines Update for Cardiopulmonary Resuscitation and Emergency Cardiovascular Care states that ultrasound may be considered during cardiopulmonary resuscitation (CPR) as long as it does not interrupt the standard ACLS protocol [8]. Its use in cardiac arrest includes identifying the underlying cause, guiding procedures, and predicting prognosis. With POCUS, cardiac motion can now be assessed during cardiac arrest without interrupting CPR. Thus far, only one systematic review and meta-analysis has examined the relationship between pseudo-PEA and return of spontaneous circulation (ROSC), neglecting other survival outcomes such as survival to hospital admission (SHA) and survival to hospital discharge (SHD) [5]. Moreover, this study focused solely on pooled risk ratios and did not include critical prognostic accuracy metrics like sensitivity, specificity, and positive and negative likelihood ratios, which provide clearer and more direct information for clinicians. Considering the increase in related research in recent years, we aim to summarize the most current evidence on the prognostic accuracy of cardiac activity on POCUS in predicting resuscitation outcomes for patients experiencing PEA.

## Materials and methods

This study adhered to the PRISMA (Preferred reporting items for systematic reviews and meta-analyses) guidelines, the Cochrane Handbook for Systematic Reviews of Diagnostic Test Accuracy, and other recognized guidelines for diagnostic accuracy reviews [9–11]. Two independent reviewers (S.-J. Z. J. and T.-H. C.) were responsible for screening studies for eligibility, extracting data, and evaluating the quality of the included studies. Any disagreements were resolved through consultation with a third reviewer (C.-C. Y.). The study protocol was registered in the PROSPERO database (CRD42024567176), and the PRISMA checklist can be found in Appendix Table 1.

### Data sources and searches

A comprehensive systematic literature search was conducted across Pubmed, Embase, and the Cochrane Central Register of Controlled Trials (CENTRAL) to identify relevant studies published up to September 23, 2024. The search strategy included medical subject headings (MeSH) and keywords related to cardiac arrest, resuscitation, PEA, ultrasonography, survival outcome, detailed in Appendix Table 2. There were no restrictions on publication date, geographical location, or language. Additionally, the reference lists of all potentially relevant studies were thoroughly reviewed.

### Study selection

Two reviewers (S.-J. Z. J. and T.-H. C.) independently assessed the eligibility of studies. They first screened the titles and abstracts of all retrieved articles to identify those potentially meeting the eligibility criteria. Articles deemed possibly eligible were then fully reviewed in full text for final determination. Disagreements were resolved by consulting a third reviewer (C.-C. Y.). To meet the qualification criteria, studies were required to be either prospective or retrospective diagnostic studies, conducted in pre-hospital or hospital settings, and to utilize transthoracic echocardiography to predict resuscitation outcomes, specifically one of the following: ROSC, SHA, or SHD. Exclusions included case reports, case series, conference abstracts, animal studies, reviews, and studies with duplicate subjects. When multiple studies used the same database, only the largest were included. Furthermore, eligible studies needed to provide sufficient data to construct a 2 × 2 table of true-positive, false-positive, true-negative, and false-negative results, either extracted directly or calculated from reported sensitivity and specificity. If these values were unavailable, the corresponding authors were contacted to request the data. The inclusion criteria's reliability was tested on a randomly selected 10% of all articles. Interobserver agreement was measured using Cohen’s kappa statistic.

### Data extraction and quality assessment

The two reviewers employed a standardized form to carry out both data extraction and risk of bias assessment. In cases of disagreement, consensus was sought or a third reviewer (C.-C. Y.) was consulted. The data collected encompassed various study details, including geographic location, eligibility criteria, patient demographics, and study settings. It also included POCUS results, resuscitation outcomes, and detailed diagnostic measures such as true-positive, false-positive, true-negative, and false-negative rates, along with the sensitivity and specificity of POCUS. The risk of bias for each study was evaluated using the Quality Assessment of Diagnostic Accuracy Studies 2 (QUADAS-2) tool [12].

### Data synthesis and analysis

We calculated the sensitivity and specificity for each study by creating a 2 × 2 contingency table. We defined positive test as cardiac activity visualized on POCUS when evaluating patients with PEA. For the meta-analysis of diagnostic accuracy, we employed a bivariate model that incorporates both fixed and random effects related to threshold and accuracy. This model facilitated the estimation of summary measures for various accuracy parameters, including sensitivity, specificity, positive and negative likelihood ratios, and diagnostic odds ratios [13]. We also applied a hierarchical summary receiver operating characteristic (HSROC) model to estimate a summary receiver operating characteristic (SROC) curve, which illustrates the relationship between sensitivity and 1-specificity [14, 15]. The 95% confidence and prediction regions around the pooled estimates were graphically depicted to illustrate the precision of these estimates (confidence ellipse) and the extent of between-study variation (prediction ellipse). Heterogeneity was assessed through visual examination of sensitivity and specificity estimates on forest plots and ROC space. We explored heterogeneity by predefined subgroup analysis using the following study-level covariates: publishing year (pre-2015 or post-2015), study design (prospective or retrospective), country (USA or non-USA), etiology (medical or trauma), and study setting (ED, ICU, or prehospital). Furthermore, we conducted sensitivity analysis using leave-one-out method by removing each study with reanalyzing the data. Publication bias was assessed using Deeks’ funnel plot of the effective sample size in conjunction with the log diagnostic odds ratio. All meta-analytic statistics were reported with their corresponding 95% confidence intervals (CIs). To evaluate the prognostic effectiveness of POCUS, we summarized our findings in a table and assessed the certainty of evidence using the GRADE approach, which rates the confidence in the accuracy of effect estimates across studies [16, 17]. Statistical and meta-analyses were conducted using STATA version 17, employing the Metadta module for summary estimates and SROC plots, and the Midas module for Deeks’ funnel plot. When the pooled study number was less than four in subgroup analyses and beyond STATA’s processing capability, summary estimates were obtained using mada package with R version 4.3.2 (R Foundation for Statistical Computing, Vienna, Austria).

## Results

### Search results

**Fig. 1:**
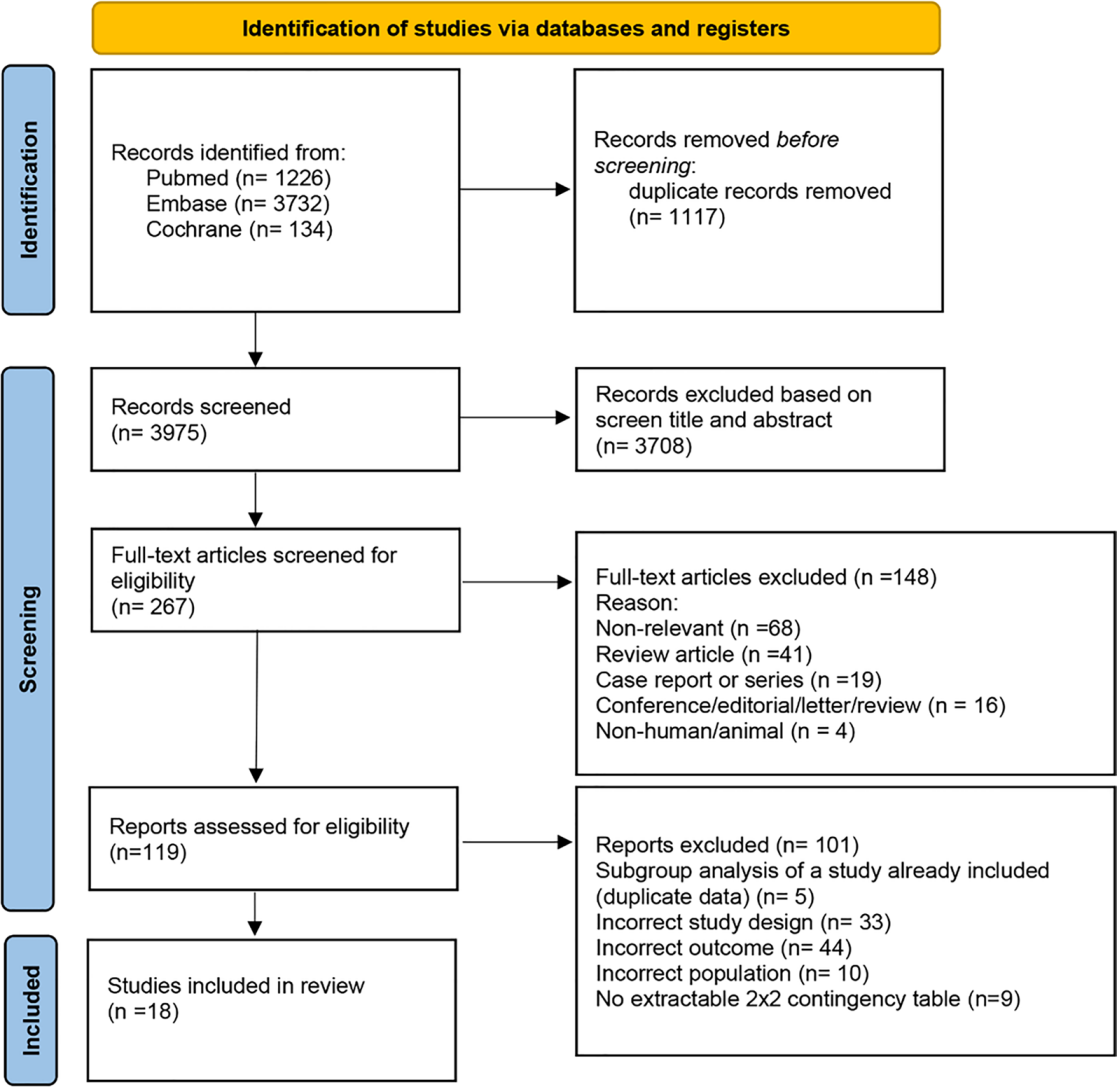
Flow chart of study identification, screening, inclusion, and exclusion for meta-analysis

Our literature search yielded 5092 articles. After removing duplicates and screening titles and abstracts, 4825 were excluded, leaving 267 articles for full-text review. Of these, 249 were excluded, and 18 articles were deemed eligible for analysis (Fig. 1). The agreement rate between the two reviewers on article selection was 90%, with a Cohen’s kappa value of k = 0.72.

### Study characteristics

Table 1 describes the characteristics of the included studies. All eligible studies were published between 2001 and 2021, featuring a median sample size of 51 (interquartile range: 33–64), and collectively encompassing a total of 1,202 patients in the final analysis. Geographically, four of the studies (22.2%) were conducted in Europe [18–21], four (22.2%) in Asia [3, 22–24], eight (44.4%) in the North America [25–32], and two (11.1%) in the South America [4, 33]. Regarding the study design, 12 studies (66.7%) were prospective cohort studies [3, 4, 19–24, 27, 30–32], and six (33.3%) were retrospective cohort studies [18, 25, 26, 28, 29, 33]. Concerning the study setting, three (16.7%) studies were conducted in the pre-hospital settings [20, 21, 30], while 15 (83.3%) were in the in-hospital setting (14 in the EDs and one in the ICU) [3, 4, 18, 19, 22–29, 31–33]. For the types of population, 10 studies included medical patients [20–22, 24, 26, 27, 30–33], three included trauma patients [25, 28, 29], while five included a mixed population [3, 4, 18, 19, 23]. In total, ten studies assessed the outcome of ROSC, with percentages varying from 18.5% to 75% and a median of 41.7% [3, 4, 19, 22, 24, 26, 27, 29, 30, 33]. Twelve studies assessed SHA, with a range of 7.0% to 58.7% and a median of 18.9% [18, 20, 21, 23, 25–32]. Finally, nine studies assessed SHD, with percentages from 0% to 19.6% and a median of 2.7% [4, 22, 25–30, 33].

**Table 1:** Main characteristics of the included studies

Author, year, country	Study design	Population (N)	Arrest setting	Study scene	Age (mean, SD)	PEA Sample size	Window	Sonographer	Training	Sonographic timing	Definition of cardiac activity	Make, model, transducer (MHz)	Outcome	
Aichinger, [20] Austria	Prospective cohort	Medical (42)	OHCA	Pre-hospital	70.3 ± 2.4	11	Subxiphoid	EP	2-h course in focused echocardiography	During a rhythm and pulse check	Any movement of myocardium	SonoSite 180(4–2-MHz) Microconvex		SHA
Beckett, [26] Canada	Retrospective cohort	Medical (180)	OHCA	ED	65.3 ± 15.0	45	Subxiphoid, parasternal long axis, or apical four chambers	EP	Not specified	During pulse and rhythm checks and necessary resuscitative procedures	Sustained coordinated contractility of the left ventricle, with visible valve movement	Machine not specified, curvilinear ± phased array		ROSC, SHA, SHD
Blaivas, [32] USA	Prospective cohort	Medical (169)	OHCA	ED	Adult (not specified)	38	Subxiphoid; if unable to obtain, use parasternal views	EP or resident	Credentialed	The duration of the pulse check	Myocardial contraction	Aloka 2000(2.5) phased arrayand curvilinear		SHA
Breitkreutz, [21] Germany	Prospective cohort	Medical (99) Traumatic (1) Shock (130)	OHCA	Pre-hospital	65 ± 19	51	Subcostal, parasternal or apical	EP	Credentialed	During an ALS conformed interruption of CPR	Coordinated cardiac activity	Modified Tringa (3.5–5) Sonosite iLook15 (4–2) curvilinear		SHA
Cebicci, [18] Turkey	Retrospective cohort	Medical (392) Traumatic (18)	OHCA or ED	ED	63.2 ± 20.7	75	Not specified	EP	Certified with good experience	Not specified	Not specified	CHISON 8500, 3,5 MHz curvilinear transducer		SHA
Chardoli, [3], Iran	Prospective cohort	Mixed (50)	OHCA	ED	58.0 ± 6.1	50	Subxiphoid	EP	Attended a teaching course to performing echocardiography	Just in the first NFI	Mechanical ventricular activities	Not specified		ROSC
Chua, [23] Singapore	Prospective cohort	Mixed (100)	OHCA	ED	68.3 ± 18.0	30	Various	EP (senior resident or above)	Passed training course (lecture, simulation, live patients, multiple choice test)	During pulse checks	Not specified	SonoSite Edge II (Fujifilm SonoSite, Inc., Bothell, WA) and Terason (Teratech Corporation, Burlington, MA) ultrasound scanners		SHA
Cureton, [28] USA	Retrospective cohort	Trauma (318)	OHCA	ED	Adult (not specified)	71	Subxiphoid	EP, Trauma surgeon, resident	Not specified	Not specified	Organized cardiac contractility, non-fibrillating movement	SonoSite MicroMaxx ultrasound scanner (SonoSite, Bothell, WA), 5-MHz curvilinear probe		SHA, SHD
Flato, [4] Brazil	Prospective cohort	Mixed (135)	ICU	In-hospital	59.8 ± 18.1	32	Subxiphoid, apical four chambers or parasternal long and short axis	Professional personnel	Had a 60-min lecture on ALS-conformed-TTE	During the intervals for rhythm check	Intrinsic movement of the myocardium coordinated with cardiac valve movement	SonoSite M Turbo,SonoSite FujiFilm Inc., Bothell, Washington, USA and a 3-MHz sector transducer		ROSC, SHD
Gaspari, [27] USA & Canada	Prospective cohort	Medical (793)	OHCA or ED	ED	64.2 ± 17.4	414	Subxiphoid or parasternal long axis	EP	Credentialed	During pauses in resuscitation	Any movement of myocardium	Not specified		ROSC
Israr, [25] USA	Retrospective cohort	Trauma (277)	OHCA	ED	43.1 ± 17.5	79	Not specified	Trauma surgeon	Not specified	Not specified	Presence of cardiac wall motion	Not specified		SHA, SHD
Jaramillo, [33] Colombia	Retrospective cohort	Medical (108)	OHCA or ED	ED	68.4 ± 13.3	56	Parasternal long axis, apical four- or five chamber view, or subxiphoid	Specialized medical staff from ED	Not specified	Not specified	Predominant ventricular and valvular activity	SonoSite M-Turbo		ROSC, SHA, SHD
Kim, [24] Korea	Prospective cohort	Medical (48)	OHCA or ED	ED	63.9 ± 14.5	8	Subcostal or parasternal	EP or resident	≧3 years experience	During pulse checks	Any ventricular, valvular or atrial motion	GE LOGIQS6(3.5) phased array		ROSC
Masoumi, [34] Iran	Prospective cohort	Medical (151)	OHCA or ED	ED	65.3 ± 11.7	62	Subxiphoid	EP	more than 6 years’ experience in emergency echocardiography	During pulse check and rhythm determination	Any visible atrial, valvular, or ventricular movement	Philips Affiniti 70 with a curved probe (2–6 MHz)		ROSC, SHD
Salen, [30] USA	Prospective cohort	Medical (102)	ED	ED	Adult (not specified)	55	Subxiphoid; the apical view as an adjunct in obese patients	EP or resident	4 h course	During the pulse check pause of the ALS	Myocardial contraction	Pie Medical200 (3.5), GERT3200 (3.5) curvilinear		SHA
Salen, [31] USA	Prospective cohort	Medical (70)	OHCA or ED	Pre-hospital and ED	Adult (not specified)	34	Subxiphoid or parasternal	EP	Not specified	Examinations during the pulse check	Any ventricular, valvular or atrial motion	Machine not specified (3.5-MHz) sector or curvilinear		ROSC, SHA, SHD
Schuster, [29] USA	Retrospective and prospective cohort	Trauma (28)	OHCA or ED	ED	Adult and pediatric 8–87 (48.6 ± 20.1)	28	Subxiphoid or parasternal	EP, surgeon, or resident under direct supervision	Complete ultrasound training	Not specified	Organized non-fibrillating cardiac activity	Philips Envisor, phased and/ or 5-MHz curvilinear probe		ROSC, SHA, SHD
Tomruk, [19] Turkey	Prospective cohort	Mixed (149)	OHCA or ED	ED	61.6 ± 17.9	64	Subxiphoid	EP	Theoretical and hands-on training in cardiac ultrasonography	During the initial assessment	Any detected motion within the heart, including atrial, valvular and/or ventricular motion	Chison 600M with a 7 MHz curvilinear transducer (Chison Medical Imaging, Wuxi City, China)		ROSC

*OHCA* Out-of-hospital cardiac arrest, *EP* Emergency physician, *ICU* Intensive care unit, *SD* Standard deviation, *ROSC* Return of spontaneous circulation, *SHA* Survival to admission, *SHD* Survival to discharge, *ED* Emergency department, *USA* United States of America, *PEA* Pulseless electrical activity, *NFI* No flow interval, ALS Advanced life support, TTE Transthoracic echocardiography

### Quality assessment

**Fig. 2:**
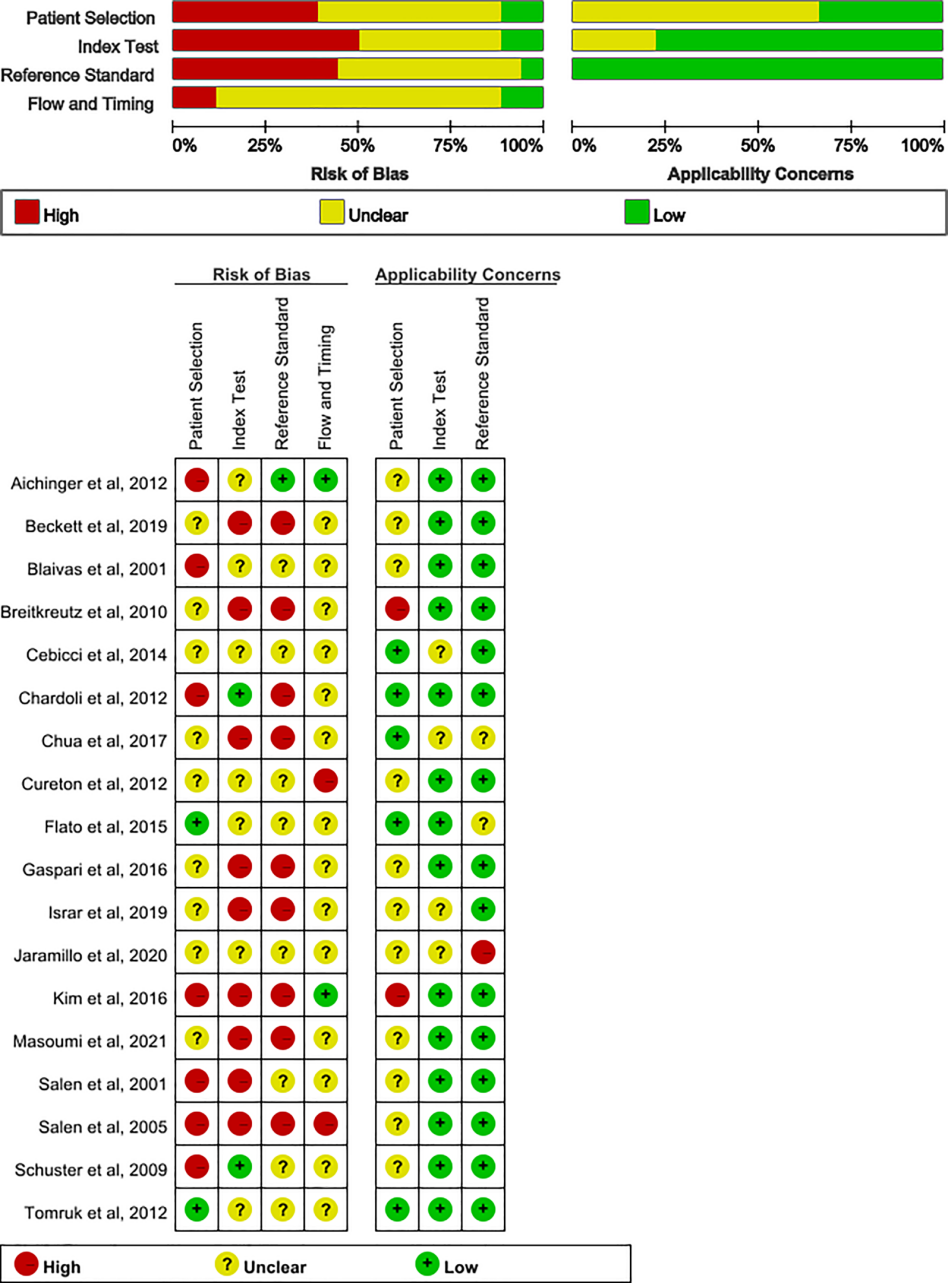
Quality assessment for 18 studies (QUADAS-2)

The overview of QUADAS-2 assessments is detailed in Appendix Table 3 and Fig. 2. Seven studies (39%) were rated as having a high risk of bias in patient selection, primarily due to the use of convenience or non-random sampling [3, 20, 24, 29–32]. In most studies (83%), unclear or high risks of bias were identified in the index test and reference standard, mainly due to the absence of pre-defined criteria for cardiac activity and a lack of a clear POCUS protocol [4, 18, 19, 21, 23–28, 30–34]. In the flow and timing domain, the absence of blinding in most studies (89%) raised concerns about the potential for decreased resuscitation efforts and self-fulfilling prophecies [3, 4, 18, 19, 21, 23, 25–28, 30–35]. Regarding applicability, twelve studies (67%) had an unclear risk of bias due to their focus on specific population [20, 24–34].

### Primary analysis of overall accuracy

**Fig. 3:**
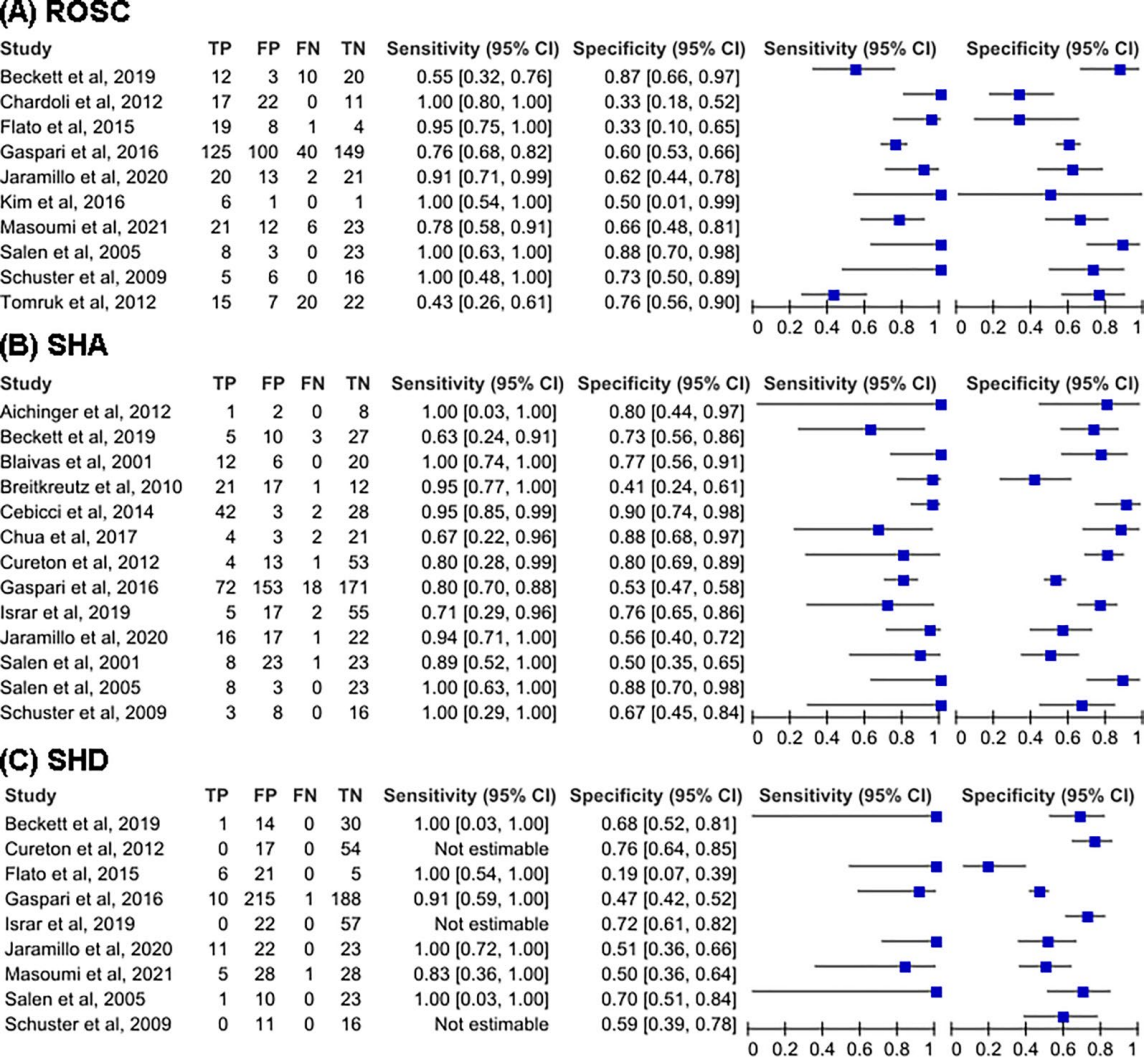
Forest plots of the sensitivity and specificity of POCUS across all included studies for the prediction of **A** ROSC, **B** SHA, and **C** SHD. *POCUS* Point-of-care ultrasound, *ROSC* Return of spontaneous circulation, *SHA* Survival to admission, *SHD* Survival to discharge, *TP* True positive, *FP* False positive, *FN* False negative, *TN* True negative, *CI* Confidence interval

**Fig. 4:**
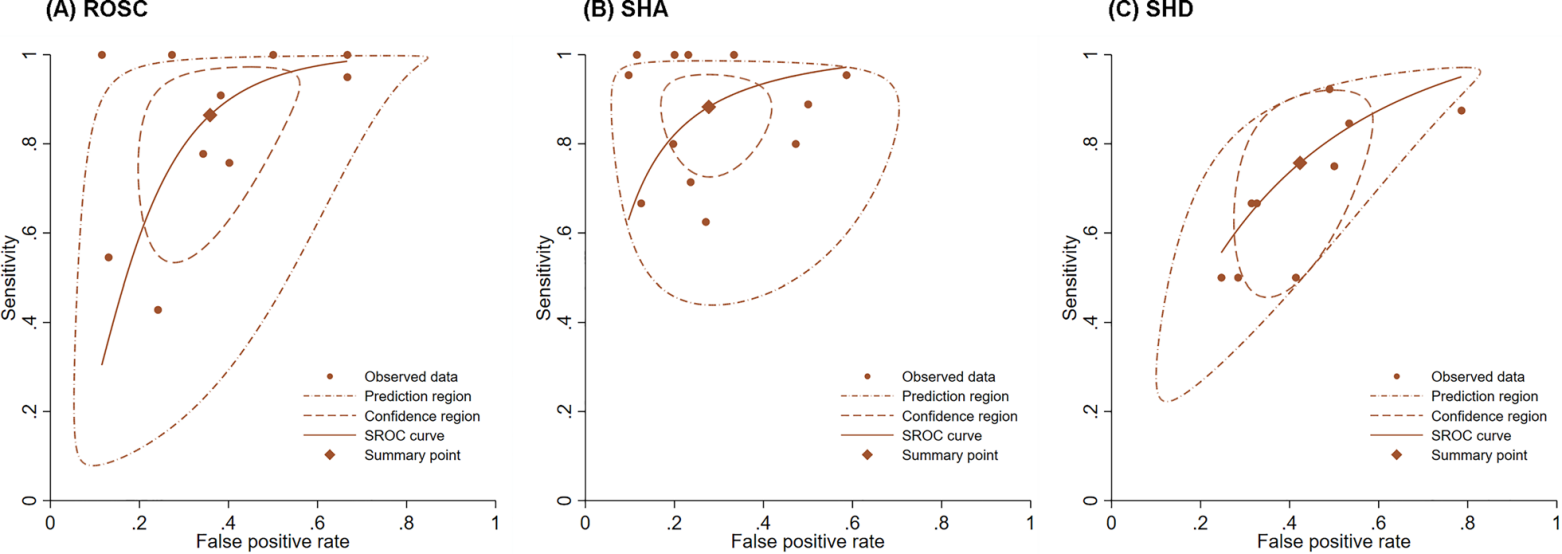
SROC curves for the diagnostic accuracy of POCUS for **A** ROSC, **B** SHA, and **C** SHD. *SROC* Summary receiver operating characteristic, *POCUS* Point-of-care ultrasound, *ROSC* Return of spontaneous circulation, *SHA* Survival to admission, *SHD* Survival to discharge;

Figure 3 shows the forest plots for the sensitivity and specificity of presence of cardiac activity on POCUS reported in the 18 included studies. For ROSC in 10 studies, the pooled sensitivity was 0.86 (95% CI 0.67–0.95), the pooled specificity was 0.64 (95% CI 0.51–0.75), and the pooled estimates of positive and negative likelihood ratios were 2.4 (95% CI 1.8–3.3) and 0.21 (95% CI 0.09–0.52), respectively. For SHA in 12 studies, the pooled sensitivity was 0.89 (95% CI 0.80–0.94), the pooled specificity was 0.73 (95% CI 0.63–0.81), and the pooled estimates of positive and negative likelihood ratios were 3.3 (95% CI 2.3–4.7) and 0.15 (95% CI 0.08–0.30), respectively. For SHD in 9 studies, the pooled sensitivity was 0.79 (95% CI 0.58–0.91), the pooled specificity was 0.58 (95% CI 0.47–0.68), and the pooled estimates of positive and negative likelihood ratios were 1.6 (95% CI 1.4–1.8) and 0.41 (95% CI 0.21–0.79), respectively (Table 2). The SROC curves, together with the bivariate summary points of specificity and sensitivity and their 95% confidence regions are shown in Fig. 4. The area under the SROC curve (AUC) was 0.79 (95% CI 0.76–0.83) for ROSC, 0.89 (95% CI 0.86–0.92) for SHA, and 0.74 (95% CI 0.57–0.78) for SHD. We calculated the posttest probabilities for both 'presence' and 'absence' of cardiac activity on POCUS for each outcome using the summary estimates across various pretest probabilities (Table 3).

**Table 2:** Summary of subgroup and sensitivity analyses of cardiac activity on POCUS in the prediction of ROSC, SHA, and SHD

Subgroup	Number of studies	Pooled sensitivity (95% CI)	Subgroup *P* value in sensitivity	Pooled specificity (95% CI)	Subgroup *P* value in specificity	Positive likelihood ratio	Negative likelihood ratio	Pooled AUC (95% CI)	Diagnostic odds ratio
*ROSC*									
Overall group	10	0.86 (0.67–0.95)	–	0.64 (0.51–0.75)	–	2.4 (1.8–3.3)	0.21 (0.09–0.52)	0.79 (0.76–0.83)	11.4 (4.2–31.2)
Publishing year									
Pre-2015	5	0.97 (0.53–1.00)	0.38	0.64 (0.40–0.82)	0.88	2.7 (1.5–4.8)	0.05 (0.01–1.17)	0.85 (0.82–0.88)	48.7 (2.3–1052)
Post-2015	5	0.75 (0.63–0.85)		0.64 (0.52–0.75)		2.1 (1.6–2.7)	0.38 (0.28–0.58)	0.75 (0.71–0.79)	5.5 (3.7–8.1)
Study design									
PS	7	0.89 (0.63–0.97)	0.85	0.59 (0.42–0.74)	0.18	2.2 (1.5–3.1)	0.19 (0.06–0.68)	0.77 (0.73–0.81)	11.2 (2.8–44.3)
RS	3	0.82 (0.51–0.95)		0.74 (0.57–0.86)		2.6 (1.9–3.8)	0.32 (0.13–0.81)	0.81 (0.64–0.89)	10.5 (4.0–27.4)
Country									
USA	3	0.87 (0.67–0.96)	0.93	0.72 (0.53–0.86)	0.38	3.1 (1.5–6.5)	0.29 (0.11–0.76)	0.86 (0.61–0.94)	15.6 (2.0–121.9)
Non-USA	7	0.82 (0.60–0.93)		0.60 (0.43–0.74)		1.8 (1.4–2.2)	0.42 (0.26–0.68)	0.74 (0.62–0.80)	5.7 (3.2–10.0)
Etiology									
Medical	6	0.80 (0.61–0.91)	0.16	0.71 (0.57–0.81)	0.85	2.7 (1.8–4.3)	0.28 (0.13–0.60)	0.82 (0.78–0.85)	9.7 (3.3–28.7)
Trauma	1	0.92 (0.52–0.99)		0.72 (0.51–0.86)		3.2 (1.6–6.5)	0.12 (0.01–1.67)	-	27.9 (1.3–580.2)
Study setting									
ED	9	0.75 (0.59–0.86)	0.25	0.65 (0.25–0.47)	0.16	2.1 (1.7–2.7)	0.42 (0.29–0.61)	0.74 (0.66–0.78)	6.0 (3.6–10.1)
ICU	1	0.95 (0.76–0.99)		0.33 (0.14–0.61)		1.4 (0.9–2.2)	0.15 (0.02–1.19)	-	9.5 (0.9–98.8)
Prehospital	0								
*SHA*									
Overall group	12	0.89 (0.80–0.94)	–	0.73 (0.63–0.81)	–	3.3 (2.3–4.7)	0.15 (0.08–0.30)	0.89 (0.86–0.92)	21.4 (8.9–51.5)
*Publishing year*									
Pre-2015	8	0.95 (0.89–0.98)	< 0.01^*^	0.74 (0.60–0.84)	0.99	3.4 (1.9–6.0)	0.06 (0.02–0.16)	0.96 (0.93–0.97)	55.0 (17.5–173)
Post-2015	4	0.72 (0.55–0.84)		0.72 (0.57–0.83)		2.5 (1.6–4.0)	0.40 (0.24–0.65)	0.77 (0.73–0.81)	6.4 (2.9–14.3)
*Study design*									
PS	7	0.90 (0.74–0.97)	0.31	0.69 (0.53–0.82)	0.07	2.9 (1.8–4.9)	0.14 (0.05–0.42)	0.90 (0.87–0.92)	21.1 (5.1–87.9)
RS	5	0.85 (0.65–0.94)		0.78 (0.71–0.84)		3.9 (2.7–5.7)	0.20 (0.08–0.51)	0.84 (0.81–0.87)	20.0 (5.6–69.3)
*Country*									
USA	6	0.90 (0.75–0.97)	0.89	0.69 (0.56–0.80)	0.25	3.0 (1.9–4.6)	0.14 (0.05–0.43)	0.83 (0.70–0.88)	20.8 (4.7–91.5)
Non-USA	6	0.88 (0.71–0.95)		0.77 (0.62–0.88)		3.9 (2.2–6.7)	0.16 (0.06–0.41)	0.90 (0.87–0.92)	24.3 (7.3–81.3)
*Etiology*									
Medical	7	0.91 (0.72–0.98)	0.65	0.66 (0.52–0.78)	0.22	2.7 (1.7–4.2)	0.13 (0.04–0.50)	0.87 (0.83–0.89)	20.0 (4.0–100.5)
Trauma	3	0.74 (0.49–0.89)		0.76 (0.69–0.82)		3.0 (2.1–4.4)	0.35 (0.16–0.77)	0.80 (0.67–0.88)	9.2 (2.9–28.8)
*Study setting*									
ED	10	0.88 (0.80–0.93)	0.30	0.75 (0.65–0.83)	0.33	3.5 (2.4–5.2)	0.16 (0.09–0.28)	0.90 (0.87–0.92)	21.7 (9.1–51.8)
ICU	0								
Prehospital	2	0.88 (0.49–0.98)		0.58 (0.23–0.87)		1.7 (1.2–2.4)	0.20 (0.05–0.74)	0.83 (0.48–0.89)	10.2 (2.1–51.0)
**SHD**									
Overall group	9	0.79 (0.58–0.91)	–	0.58 (0.47–0.68)	–	1.6 (1.4–1.8)	0.41 (0.21–0.79)	0.74 (0.57–0.78)	5.1 (2.1–12.5)
*Publishing year*									
Pre-2015	4	0.74 (0.28–0.95)	0.71	0.57 (0.31–0.79)	0.99	1.2 (1.0–1.6)	0.57 (0.19–1.72)	0.70 (0.40–0.76)	3.4 (0.6–19.5)
Post-2015	5	0.81 (0.43–0.93)		0.57 (0.46–0.67)		1.7 (1.5–2.1)	0.34 (0.15–0.78)	0.74 (0.59–0.84)	5.9 (2.1–16.9)
*Study design*									
PS	4	0.85 (0.62–0.95)	0.57	0.47 (0.34–0.71)	0.04^*^	1.5 (1.2–1.9)	0.35 (0.14–0.85)	0.77 (0.52–0.85)	4.8 (1.6–14.4)
RS	5	0.73 (0.31–0.94)		0.66 (0.58–0.75)		2.0 (1.5–2.6)	0.50 (0.19–1.31)	0.72 (0.49–0.76)	5.8 (1.2–27.8)
*Country*									
USA	3	0.62 (0.07–0.83)	0.35	0.68 (0.60–0.76)	0.21	2.1 (1.0–4.6)	0.64 (0.19–2.13)	0.70 (0.41–0.74)	3.3 (0.4–27.5)
Non-USA	6	0.84 (0.63–0.94)		0.53 (0.38–0.67)		1.6 (1.3–1.9)	0.34 (0.16–0.75)	0.77 (0.56–0.80)	5.6 (2.1–15.2)
*Etiology*									
Medical	5	0.84 (0.64–0.94)	0.20	0.55 (0.46–0.64)	0.03^*^	1.8 (1.5–2.1)	0.30 (0.13–0.71)	0.78 (0.50–0.86)	6.3 (2.2–17.9)
Trauma	3	0.50 (0.09–0.91)		0.71 (0.64–0.77)		1.6 (0.5–5.2)	0.73 (0.23–2.30)	0.56 (0.49–0.76)	2.3 (0.2–22.3)
*Study setting*									
ED	8	0.76 (0.53–0.90)	0.40	0.61 (0.52–0.69)	< 0.01^*^	1.8 (1.5–2.1)	0.41 (0.21–0.82)	0.73 (0.61–0.78)	5.3 (2.1–13.7)
ICU	1	0.93 (0.56–0.99)		0.20 (0.09–0.39)		1.2 (0.9–1.5)	0.35 (0.02–5.61)		3.3 (0.2–68.5)
Prehospital	0								

*ROSC* Return of spontaneous circulation, *SHA* Survival to admission, *SHD* Survival to discharge, *ED* Emergency department, *ICU* Intensive care unit, *POCUS* Point-of-care ultrasound, *PS* Prospective, *RS* Retrospective, *USA* United States of America

^*^P < 0.05

**Table 3:** Posttest probabilities for ROSC, SHA, and SHD for a sample of population prevalence, determined using POCUS

Pretest probability	Posttest probability after the presence of cardiac activity on POCUS	Posttest probability after the absence of cardiac activity on POCUS	False positive^*^	False negative^*^
*ROSC (sensitivity: 0.86, specificity: 0.64)*				
0.1	0.21	0.02	324	14
0.25	0.44	0.07	270	35
0.5	0.71	0.17	180	70
0.75	0.88	0.39	90	105
*SHA (sensitivity: 0.89, specificity: 0.73)*				
0.1	0.27	0.02	243	11
0.25	0.52	0.05	203	28
0.5	0.77	0.13	135	55
0.75	0.91	0.31	68	83
*SHD (sensitivity: 0.79, specificity: 0.58)*				
0.1	0.15	0.04	378	21
0.25	0.35	0.12	315	53
0.5	0.62	0.29	210	105
0.75	0.83	0.55	105	158

*ROSC* Return of spontaneous circulation, *SHA* Survival to admission, *SHD* Survival to discharge, *POCUS* Point-of-care ultrasound

^*^Number of false positives and negatives in 1000 hypothetical cases

### Subgroup and sensitivity analyses

There was significant heterogeneity observed in the included studies, mostly indicated by the broad range of specificity estimates. To explore potential sources of the heterogeneity, we conducted subgroup analyses (Table 2). For SHA, studies publishing after 2015 showed significantly lower sensitivity compared with those publishing before 2015 (0.72; 95% CI 0.55–0.84 vs 0.95; 95% CI 0.89–0.98; *p* < 0.01). For SHD, studies with prospective design showed significantly lower specificity compared with those with retrospective design (0.47; 95% CI 0.34–0.71 vs 0.66; 95% CI 0.58–0.75; *p* = 0.04), studies enrolling medical patients showed significantly lower specificity compared with those enrolling trauma patients (0.47; 95% CI 0.34–0.71 vs 0.66; 95% CI 0.58–0.75; *p* = 0.04), and studies enrolling ED patients showed significantly higher specificity compared with those enrolling ICU patients (0.61; 95% CI 0.52–0.69 vs 0.20; 95% CI 0.09–0.39; *p* < 0.01). Sensitivity analysis demonstrated the pooled AUC did not significantly differ when removing each study for each outcome (Appendix Table 4).

### Publication bias

**Fig. 5:**
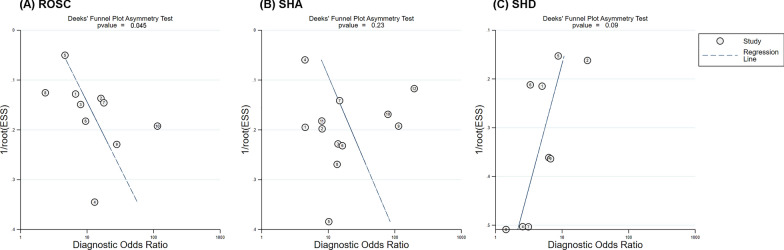
Deeks' funnel plot (asymmetry test) for **A** ROSC, **B** SHA and **C** SHD. *ROSC* Return of spontaneous circulation, *SHA* Survival to admission; *SHD* Survival to discharge

Ten studies assessing ROSC indicated a significant publication bias (*p* = 0.045), while 12 studies assessing SHA and nine assessing SHD showed no significant publication bias (*p* = 0.23 and *p* = 0.09) (Fig. 5).

### Certainty of evidence

The certainty of evidence for POCUS in identifying ROSC was rated as 'very low' for both sensitivity and specificity. For SHA, the certainty was rated as 'moderate' for sensitivity and 'low' for specificity. For SHD, it was rated as 'moderate' for sensitivity and 'very low' for specificity. These ratings were influenced by risks of bias, inconsistency, and publication bias. Detailed evaluations are provided in the GRADE evidence profile in Appendix Table 5.

## Discussion

### Summary of the main results

To our knowledge, this is the first systematic review and meta-analysis specifically focusing on patients presenting with PEA rhythm to assess the prognostic accuracy of POCUS. Eighteen studies, including a total of 1,202 patients, were analyzed, and our results indicate that the presence of cardiac activity during CPR demonstrates high prognostic performance for predicting SHA, with pooled sensitivity, specificity and AUC as 0.89 (95% CI 0.80–0.94), 0.73 (95% CI 0.63–0.81), and 0.89 (95% CI 0.86–0.92), respectively. The effectiveness of a prognostic tool for cardiac arrest patients depends on balancing the risk of prematurely terminating resuscitation with the risk of prolonging unnecessary efforts. These findings suggest that POCUS can be a valuable tool for assessing survival potential during CPR and may assist in decisions regarding the termination of resuscitation (TOR). In a hypothetical cohort of 1,000 patients with a median pretest probability of 20% for SHA, the use of POCUS alone would result in 22 false negatives (patients who survive to hospital admission despite no cardiac activity detected on POCUS) and 216 false positives (patients who do not survive to hospital admission despite cardiac activity detected on POCUS). A negative POCUS result would yield a posttest probability of 4%, while a positive result would increase the posttest probability to 55%. While POCUS alone may lack sufficient accuracy to guide early decisions for TOR, it can serve as a vital component of a multimodal approach by offering valuable information that complements other clinical assessments.

### Suboptimal performance of POCUS in predicting ROSC and SHD

Compared to SHA, POCUS demonstrates only moderate prognostic accuracy for ROSC and SHD. Unlike SHA, which generally has a consistent definition, the variability in ROSC definitions likely accounts for its lower prognostic accuracy. For example, Tomruk et al. defined ROSC as the presence of a sustained palpable pulse and measurable blood pressure for at least 20 min [25], whereas Chardoli et al. defined it as a palpable pulse and detectable blood pressure for at least 10 s [3]. This inconsistency in definitions contributes to heterogeneity and diminishes the predictive ability of POCUS for ROSC. Regarding SHD, the decline in prognostic performance may be attributed to factors such as patients’ underlying health conditions, complications arising from invasive treatments and procedures, and comorbidities acquired during hospitalization. These factors may not directly correlate with the initial POCUS findings in prehospital or ED settings.

### Heterogeneity and subgroup analyses

A substantial heterogeneity was observed among the included studies, which may be attributed to factors such as differences in POCUS protocols, the timing of POCUS performance, the number of examinations conducted, the types of views obtained, and varying definitions of cardiac activity and ROSC. The definition of cardiac activity varied across the included studies, ranging from unspecified descriptions such as "coordinated cardiac activity" to more detailed operational definitions like "sustained coordinated contractility of the left ventricle, with visible valve movement." This inconsistency aligns with findings from a prospective survey study by Hu et al., which involved faculty, fellows, and resident physicians specializing in emergency medicine, critical care, and cardiology [36]. Participants in this study, shown sonographic video clips from 15 cases of cardiac arrest, demonstrated only moderate agreement (α = 0.47) on what constituted cardiac standstill. The clips that garnered the least consensus were characterized by one or more of the following: valve flutter, mechanical ventilation, weak myocardial contraction, or profound bradycardia. We encourage future studies to adopt a clear and consistent definition of cardiac activity to standardize the use of POCUS at the bedside.

We performed various subgroup analyses to identify potential sources of heterogeneity. In the subgroup analysis of the SHD group, retrospective studies showed a higher pooled specificity compared to prospective studies. These differences may be attributed to variations in methodological analysis and data collection. Retrospective studies obtain data by reviewing historical medical records and collecting information based on their research design, which can potentially introduce record bias. A higher pooled specificity was observed in traumatic cardiac arrest compared to medical cardiac arrest, highlighting differences in their pathophysiology. In traumatic cases, POCUS often acts as a marker of shock severity, with the absence of cardiac activity indicating catastrophic and typically irreversible injuries. For SHD, studies conducted in the ICU revealed lower pooled specificity compared to ED studies. However, only one ICU study was available, involving 27 pseudo-EMD patients, of whom 19 achieved ROSC and six survived to hospital discharge [4]. These findings may underscore differences between ICU and ED patient populations, with ICU patients tending to have more complex conditions and additional comorbidities [37–39]. In 2015, the AHA issued a Class IIB recommendation for the use of ultrasonography in cardiac arrest [8]. Notably, studies conducted before 2015 demonstrated higher pooled sensitivity for SHA compared to those conducted afterward. The reason for this discrepancy is unclear but is likely due to a combination of factors, including differences in study methodologies, operator expertise, technological advancements, and evolving clinical practices.

### Strengths and limitations of the review

The most recent meta-analysis specifically examining PEA patients was conducted in 2018 by Wu et al. [5]. They concluded that bedside ultrasound is valuable for predicting ROSC and aiding decisions to terminate resuscitation. However, their study reported only pooled risk ratios, omitting sensitivity and specificity—critical metrics for clinical decision-making. Furthermore, some studies included in their analysis examined different outcomes, such as SAH or SHD, but categorized them all as ROSC, raising concerns about result validity. Our systematic review and meta-analysis improve validity and applicability by (1) analyzing diverse resuscitation outcomes using the bivariate model; (2) conducting detailed QUADAS-2 assessments and evidence certainty evaluations of included studies for greater transparency and rigor; (3) performing additional subgroup analyses to address potential heterogeneity; and (4) utilizing sensitivity analyses to ensure result robustness.

There are several limitations in our study. First, although we employed a rigorous search strategy without language restrictions, we may have missed relevant articles. Second, all included studies were observational cohort studies, and the lack of blinding in most studies raises concerns about biasing survival outcomes. For example, the absence of blinding regarding POCUS results to the resuscitation team could lead to premature termination of resuscitation, potentially inflating the diagnostic accuracy of POCUS in cardiac arrest patients by reinforcing the association between cardiac standstill and mortality. Evidence suggests that patients with observed cardiac activity during resuscitation are more likely to receive prolonged resuscitation efforts, including more frequent endotracheal intubation and epinephrine administration [27, 40]. Third, substantial heterogeneity in sensitivity and specificity estimates, as revealed by subgroup analyses, highlights how differences in study designs, populations, and settings may limit the generalizability of the pooled results. Additionally, the accuracy of POCUS might vary with operator experience, a factor not systematically examined in this review due to limited reporting [41]. Fourth, significant publication bias in studies assessing ROSC diminishes the reliability of the pooled accuracy metrics for this outcome. Fifth, many studies in the review exhibited high or unclear risk of bias, especially in patient selection and the index test, due to the lack of universally accepted criteria for cardiac activity and variability in POCUS protocols. Lastly, various other factors, such as a patient’s underlying health conditions (e.g., cancer status), the etiology of the arrest, downtime before CPR initiation, and the quality and duration of CPR, may influence outcomes [42]. However, incomplete data on these variables precluded further analyses to assess their impact.

## Conclusion

This systematic review and meta-analysis shows that among 1,202 patients across 18 studies, POCUS exhibits high prognostic accuracy for SHA and moderate accuracy for ROSC and SHD in PEA patients. POCUS alone does not appear to provide adequate accuracy for guiding early TOR treatment decisions. Instead, it should serve as a complement to, rather than a replacement for, comprehensive clinical evaluations. Future research should explore strategies like integrating POCUS with traditional prognostic variables to develop clinical scoring systems that enhance the accuracy of resuscitation outcome predictions.

## Supplementary Information

Additional file 1

Additional file 2

## Data Availability

This review uses summary data from cited publications, which are retrievable by referring to the original manuscripts.

## Abbreviations

PEA: Pulseless electrical activity
POCUS: Point-of-care ultrasound
PLR: Positive likelihood ratio
NLR: Negative likelihood ratio
SROC: Summary receiver operating characteristic curve
EMD: Electromechanical dissociation
ROSC: Return of spontaneous circulation
PRISMA: Preferred reporting items for systematic reviews and meta-analyses
TOR: Termination of resuscitation
