# Supplementary material for: Prognostic accuracy of point-of-care ultrasound in patients with pulseless electrical activity: a systematic review and meta-analysis
Source: Scand J Trauma Resusc Emerg Med. 2025 Feb 10;33:27. doi: 10.1186/s13049-025-01327-0 (PMC11812266; doi:10.1186/s13049-025-01327-0)
Supplement: Supplementary file 2 — Additional file 2 [file 13049_2025_1327_MOESM2_ESM.docx]

# Appendix Table 1. PRISMA checklist

| **Section/topic** | **#** | | **PRISMA-DTA Checklist Item** | | **Reported on page #** | |  |
| --- | --- | --- | --- | --- | --- | --- | --- |
| **TITLE / ABSTRACT** | | | | | 1 | |  |
| Title | 1 | | Identify the report as a systematic review (+/- meta-analysis) of diagnostic test accuracy (DTA) studies. | | 1 | |  |
| Abstract | 2 | | Abstract: See PRISMA-DTA for abstracts. | | 2-3 | |  |
| **INTRODUCTION** | | | | | 4 | |  |
| Rationale | 3 | | Describe the rationale for the review in the context of what is already known. | | 4 | |  |
| Clinical role of index test | D1 | | State the scientific and clinical background, including the intended use and clinical role of the index test, and if applicable, the rationale for minimally acceptable test accuracy (or minimum difference in accuracy for comparative design). | | 4 | |  |
| Objectives | 4 | | Provide an explicit statement of question(s) being addressed in terms of participants, index test(s), and target condition(s). | | 4-5 | |  |
| **METHODS** | | | | | 5 | |  |
| Protocol and registration | 5 | | Indicate if a review protocol exists, if and where it can be accessed (e.g., Web address), and, if available, provide registration information including registration number. | | 5 | |  |
| Eligibility criteria | 6 | | Specify study characteristics (participants, setting, index test(s), reference standard(s), target condition(s), and study design) and report characteristics (e.g., years considered, language, publication status) used as criteria for eligibility, giving rationale. | | 5 | |  |
| Information sources | 7 | | Describe all information sources (e.g., databases with dates of coverage, contact with study authors to identify additional studies) in the search and date last searched. | | 5 | |  |
| Search | 8 | | Present full search strategies for all electronic databases and other sources searched, including any limits used, such that they could be repeated. | | 5 | |  |
| Study selection | 9 | | State the process for selecting studies (i.e., screening, eligibility, included in systematic review, and, if applicable, included in the meta-analysis). | | 6 | |  |
| Data collection process | 10 | | Describe method of data extraction from reports (e.g., piloted forms, independently, in duplicate) and any processes for obtaining and confirming data from investigators. | | 6 | |  |
| Definitions for data extraction | 11 | | Provide definitions used in data extraction and classifications of target condition(s), index test(s), reference standard(s) and other characteristics (e.g. study design, clinical setting). | | 6-7 | |  |
| Risk of bias and applicability | 12 | | Describe methods used for assessing risk of bias in individual studies and concerns regarding the applicability to the review question. | | 6-7 | |  |
| Diagnostic accuracy measures | 13 | | State the principal diagnostic accuracy measure(s) reported (e.g. sensitivity, specificity) and state the unit of assessment (e.g. per-patient, per-lesion). | | 7-8 | |  |
| Synthesis of results | 14 | | Describe methods of handling data, combining results of studies and describing variability between studies. This could include, but is not limited to: a) handling of multiple definitions of target condition. b) handling of multiple thresholds of test positivity, c) handling multiple index test readers, d) handling of indeterminate test results, e) grouping and comparing tests, f) handling of different reference standards | | 7-8 | |  |
| Section/topic | # | | PRISMA-DTA Checklist Item | | Reported on page # | |  |
| Meta-analysis | D2 | | Report the statistical methods used for meta-analyses, if performed. | | 7-8 | |  |
| Additional analyses | 16 | | Describe methods of additional analyses (e.g., sensitivity or subgroup analyses, meta-regression), if done, indicating which were pre-specified. | | 7-8 | |  |
| **RESULTS** | | | | | | 8 | |
| Study selection | | 17 | | Provide numbers of studies screened, assessed for eligibility, included in the review (and included in meta-analysis, if applicable) with reasons for exclusions at each stage, ideally with a flow diagram. | | 8 | |
| Study characteristics | | 18 | | For each included study provide citations and present key characteristics including: a) participant characteristics (presentation, prior testing), b) clinical setting, c) study design, d) target condition definition, e) index test, f) reference standard, g) sample size, h) funding sources | | 8-9 | |
| Risk of bias and applicability | | 19 | | Present evaluation of risk of bias and concerns regarding applicability for each study. | | 9 | |
| Results of individual studies | | 20 | | For each analysis in each study (e.g. unique combination of index test, reference standard, and positivity threshold) report 2x2 data (TP, FP, FN, TN) with estimates of diagnostic accuracy and confidence intervals, ideally with a forest or receiver operator characteristic (ROC) plot. | | 10 | |
| Synthesis of results | | 21 | | Describe test accuracy, including variability; if meta-analysis was done, include results and confidence intervals. | | 10 | |
| Additional analysis | | 23 | | Give results of additional analyses, if done (e.g., sensitivity or subgroup analyses, meta-regression; analysis of index test: failure rates, proportion of inconclusive results, adverse events). | | 10-11 | |
| **DISCUSSION** | | | | | | 11 | |
| Summary of evidence | | 24 | | Summarize the main findings including the strength of evidence. | | 11-14 | |
| Limitations | | 25 | | Discuss limitations from included studies (e.g. risk of bias and concerns regarding applicability) and from the review process (e.g. incomplete retrieval of identified research). | | 14-15 | |
| Conclusions | | 26 | | Provide a general interpretation of the results in the context of other evidence. Discuss implications for future research and clinical practice (e.g. the intended use and clinical role of the index test). | | 15 | |
| **FUNDING** | | | | | | 17 | |
| Funding | | 27 | | For the systematic review, describe the sources of funding and other support and the role of the funders. | | 17 | |

# Appendix Table 2. Electronic search strategies

1. PubMed

Date limits: 1979 to September 23, 2024

|  | Search strategy | Results |
| --- | --- | --- |
| 1 | ACLS[Title/Abstract] | 1,348 |
| 2 | Cardiac contraction[Title/Abstract] | 1,351 |
| 3 | Cardiac activity[Title/Abstract] | 2,978 |
| 4 | Cardiac movement[Title/Abstract] | 73 |
| 5 | cardiac massage[Title/Abstract] | 1159 |
| 6 | CPR[MeSH Terms] | 23,114 |
| 7 | Cardiopulmonary resuscitation[MeSH Terms] | 23,114 |
| 8 | Cardiopulmonary resuscitation[Title/Abstract] | 20,327 |
| 9 | cardiac arrest[Title/Abstract] | 45,910 |
| 10 | chest compression[Title/Abstract] | 2,983 |
| 11 | electromechanical dissociation[Title/Abstract] | 459 |
| 12 | heart arrest[MeSH Terms] | 57,804 |
| 13 | PEA[MeSH Terms] | 5,245 |
| 14 | pulseless electrical activity[Title/Abstract] | 1,160 |
| 15 | Resuscitation[Title/Abstract] | 71,179 |
| 16 | 1 or 2 or 3 or 4 or 5 or 6 or 7 or 8 or 9 or 10 or 11 or 12 or 13 or 14 or 15 | 141882 |
| 17 | ultrasonography[MeSH Terms] | 495,080 |
| 18 | bedside ultrasound[Title/Abstract] | 1,323 |
| 19 | sonography[Title/Abstract] | 35,923 |
| 20 | echocardiography[MeSH Terms] | 151,099 |
| 21 | cardiac sonography[Title/Abstract] | 56 |
| 22 | cardiac ultrasonography[Title/Abstract] | 284 |
| 23 | transthoracic echocardiography[Title/Abstract] | 14,710 |
| 24 | transthoracic echocardiogram[Title/Abstract] | 2,772 |
| 25 | TTE[Title/Abstract] | 5,997 |
| 26 | echocardi*[Title/Abstract] | 188,180 |
| 27 | POCUS[Title/Abstract] | 2,677 |
| 28 | Point-of-care ultras*[Title/Abstract] | 4,929 |
| 29 | 17 or 18 or 19 or 20 or 21 or 22 or 23 or 24 or 25 or 26 or 27 or 28 | 587,114 |
| 30 | ROSC[Title/Abstract] | 3,439 |
| 31 | Return of spontaneous circulation[Title/Abstract] | 4,747 |
| 32 | predict*[Title/Abstract] | 2,174,577 |
| 33 | survival[Title/Abstract] | 1,237,585 |
| 34 | outcome[Title/Abstract] | 1,329,002 |
| 35 | 30 or 31 or 32 or 33 or 34 | 4,152,123 |
| 36 | 16 and 29 and 35 | 1226 |

1. EMBASE

Date limits: 1966 to September 23, 2024

|  | Search strategy | Results |
| --- | --- | --- |
| 1 | 'advanced cardiac life support'/exp | 846 |
| 2 | 'cardiac contraction':ti,ab OR 'cardiac activity':ti,ab OR 'cardiac movement':ti,ab OR 'cardiac massage':ti,ab OR 'cardiopulmonary resuscitation':ti,ab OR 'cardiac arrest':ti,ab OR 'emd':ti,ab OR 'electromechanical dissociation':ti,ab OR 'heart arrest':ti,ab OR 'heart massage':ti,ab OR 'pea':ti,ab OR 'pulseless electrical activity':ti,ab OR 'resuscitation':ti,ab | 185,029 |
| 3 | 'echocardiography'/exp | 468,133 |
| 4 | 'ultrasonography':ti,ab OR 'bedside ultrasound':ti,ab OR 'sonography':ti,ab OR 'cardiac sonography':ti,ab OR 'cardiac ultrasound':ti,ab OR 'transthoracic echocardiography':ti,ab OR 'transthoracic echocardiogram':ti,ab OR 'tte':ti,ab OR 'echocardi*':ti,ab OR 'pocus':ti,ab OR 'point-of-care ultras*':ti,ab | 534,755 |
| 5 | 'survival'/exp | 1,527,213 |
| 6 | 'rosc':ab,ti OR 'return of spontaneous circulation':ab,ti OR 'predict*':ab,ti OR 'outcome':ab,ti | 4,552,289 |
| 7 | 1 or 2 | 185,332 |
| 8 | 3 or 4 | 715,596 |
| 9 | 5 or 6 | 5,591,246 |
| 10 | 7 AND 8 AND 9 | 3,732 |

(C) Cochrane library

Date limits: 1995 to September 23, 2024

|  | Search strategy | Results |
| --- | --- | --- |
| 1 | Advanced Cardiac Life Support: ti,ab,kw | 473 |
| 2 | pulseless electrical activity: ti,ab,kw | 85 |
| 3 | Cardiopulmonary resuscitation: ti,ab,kw | 2999 |
| 4 | cardiac arrest: ti,ab,kw | 5437 |
| 5 | heart arrest: ti,ab,kw | 5098 |
| 6 | Resuscitation: ti,ab,kw | 9347 |
| 7 | echoc*: ti,ab,kw | 17514 |
| 8 | ultras*: ti,ab,kw | 60690 |
| 9 | Point-of-care ultras*: ti,ab,kw | 484 |
| 10 | ROSC: ti,ab,kw | 488 |
| 11 | Return of spontaneous circulation: ti,ab,kw | 764 |
| 12 | predict*: ti,ab,kw | 120589 |
| 13 | # survival: ti,ab,kw | 132843 |
| 14 | outcome: ti,ab,kw | 689805 |
| 15 | #1 OR #2 OR #3 OR #4 OR #5 OR #6 | 13607 |
| 16 | #7 OR #8 OR #9 | 76600 |
| 17 | #10 OR #11 OR #12 OR #13 OR #14 | 826326 |
| 18 | #15 AND #16 AND #17 | 134 |

# Appendix Table 3. Risk of bias and concerns of applicability for the 18 studies included in this review

| **Study** | **Patient Selection** | **Index test(s)** | **Reference Standard** | **Flow and timing** |
| --- | --- | --- | --- | --- |
| Aichinger, 2012 | Risk of Bias: high   - Non-consecutive patients - Prospective - Avoided inappropriate exclusion   Concerns about applicability: unclear   - Limited to medical population | Risk of Bias: unclear   - It is unclear whether the index test results were interpreted independently of the reference standard results   Concerns about applicability: low   - Index test conduct and execution clear - Index test interpretation clear | Risk of Bias: low   - Outcome assessment independent of test - Adequate descriptions of reference standards   Concerns about applicability: low   - Relevant outcomes | Risk of Bias: low   - Appropriate interval between index test and reference standard - All patient’s outcome assessed - All patients received the same reference standards - Timing of index tests clear |
| Beckett, 2019 | Risk of Bias: unclear   - Selection process unclear - Retrospective - Avoided inappropriate exclusion   Concerns about applicability: unclear   - Limited to medical population | Risk of Bias: high   - The index test results were not interpreted without knowledge of the reference standard results   Concerns about applicability: low   - Index test conduct and execution clear - Index test interpretation clear | Risk of Bias: high   - The reference standard results were not interpreted without knowledge of the index test results - Adequate descriptions of reference standards   Concerns about applicability: low   - Relevant outcomes | Risk of Bias: unclear   - The interval between the index test and the reference standard was not clearly defined - All patient’s outcome assessed - All patients received the same reference standards - Timing of index tests clear |
| Blaivas, 2001 | Risk of Bias: high   - Non-consecutive patients - Prospective - Avoided inappropriate exclusion   Concerns about applicability: unclear   - Limited to medical population | Risk of Bias: unclear   - It is unclear whether the index test results were interpreted independently of the reference standard results   Concerns about applicability: low   - Index test conduct and execution clear - Index test interpretation clear | Risk of Bias: unclear   - It is unclear whether the reference standard results were interpreted without knowledge of the index test results - Adequate descriptions of reference standards   Concerns about applicability: low   - Relevant outcomes | Risk of Bias: unclear   - The interval between the index test and the reference standard was not clearly defined - All patients received same reference standards - All patient’s outcome assessed - Timing of index tests clear |
| Breitkreutz, 2010 | Risk of Bias: unclear   - Selection process unclear - Prospective - Avoided inappropriate exclusion   Concerns about applicability: low   - Appropriate study population | Risk of Bias: high   - The index test results were not interpreted without knowledge of the reference standard results   Concerns about applicability: low   - Index test conduct and execution clear - Index test interpretation clear | Risk of Bias: high   - The reference standard results were not interpreted without knowledge of the index test results - Adequate descriptions of reference standards   Concerns about applicability: low   - Relevant outcomes | Risk of Bias: unclear   - The interval between the index test and the reference standard was not clearly defined - All patients received same reference standards - All patient’s outcome assessed - Timing of index tests clear |
| Cebicci, 2014 | Risk of Bias: unclear   - Selection process unclear - Retrospective - Avoided inappropriate exclusion   Concerns about applicability: low   - Appropriate study population | Risk of Bias: unclear   - It is unclear whether the index test results were interpreted independently of the reference standard results   Concerns about applicability: unclear   - The manner in which the index test was conducted and performed was not specified - The definition of cardiac activity was not clearly specified | Risk of Bias: unclear   - It is unclear whether the reference standard results were interpreted without knowledge of the index test results - Adequate descriptions of reference standards   Concerns about applicability: low   - Relevant outcomes | Risk of Bias: unclear   - The interval between the index test and the reference standard was not clearly defined - It is unclear whether all patients received the same reference standards - All patient’s outcome assessed - Timing of index tests unclear |
| Chardoli, 2012 | Risk of Bias: high   - Convenience sample selection - Prospective - No clear exclusion criteria     Concerns about applicability: low   - Appropriate study population | Risk of Bias: low   - Index tests interpreted without knowledge of outcome   Concerns about applicability: low   - Index test conduct and execution clear - Index test interpretation clear | Risk of Bias: high   - The reference standard results were not interpreted without knowledge of the index test results - Adequate descriptions of reference standards   Concerns about applicability: low   - Relevant outcomes | Risk of Bias: unclear   - The interval between the index test and the reference standard was not clearly defined - All patients received same reference standards - All patient’s outcome assessed - Timing of index tests clear |
| Chua, 2017 | Risk of Bias: unclear   - Selection process unclear - Prospective - Avoided inappropriate exclusion   Concerns about applicability: low   - Appropriate study population | Risk of Bias: high   - The index test results were not interpreted without knowledge of the reference standard results   Concerns about applicability: unclear   - Index test conduct and execution clear - The definition of cardiac activity was not clearly specified | Risk of Bias: high   - The reference standard results were not interpreted without knowledge of the index test results - Adequate descriptions of reference standards   Concerns about applicability: low   - Relevant outcomes | Risk of Bias: unclear   - The interval between the index test and the reference standard was not clearly defined - All patients received same reference standards - All patient’s outcome assessed - Timing of index tests clear |
| Cureton, 2012 | Risk of Bias: unclear   - Selection process unclear - Retrospective - No clear exclusion criteria   Concerns about applicability: unclear   - Limited to trauma population | Risk of Bias: unclear   - It is unclear whether the index test results were interpreted independently of the reference standard results   Concerns about applicability: low   - Index test conduct and execution clear - Index test interpretation clear | Risk of Bias: unclear   - It is unclear whether the reference standard results were interpreted without knowledge of the index test results - Adequate descriptions of reference standards   Concerns about applicability: low   - Relevant outcomes | Risk of Bias: high   - The interval between the index test and the reference standard was not clearly defined - All patients received same reference standards - Not all patient’s outcome assessed - Timing of index tests clear unclear |
| Flato, 2015 | Risk of Bias: low   - Selection process clear (Consecutive) - Prospective - Avoided inappropriate exclusion   Concerns about applicability: low   - Appropriate study population | Risk of Bias: unclear   - It is unclear whether the index test results were interpreted independently of the reference standard results   Concerns about applicability: low   - Index test conduct and execution clear - Index test interpretation clear | Risk of Bias: unclear   - It is unclear whether the reference standard results were interpreted without knowledge of the index test results - Adequate descriptions of reference standards   Concerns about applicability: low   - Relevant outcomes | Risk of Bias: unclear   - The interval between the index test and the reference standard was not clearly defined - All patients received same reference standards - All patient’s outcome assessed - Timing of index tests clear |
| Gaspari, 2016 | Risk of Bias: unclear   - Selection process unclear - Prospective - Avoided inappropriate exclusion   Concerns about applicability: unclear   - Limited to medical population | Risk of Bias: high   - The index test results were not interpreted without knowledge of the reference standard results   Concerns about applicability: low   - Index test conduct and execution clear - Index test interpretation clear | Risk of Bias: high   - The reference standard results were not interpreted without knowledge of the index test results - Adequate descriptions of reference standards   Concerns about applicability: low   - Relevant outcomes | Risk of Bias: unclear   - The interval between the index test and the reference standard was not clearly defined - All patients received same reference standards - All patient’s outcome assessed - Timing of index tests clear |
| Israr, 2019 | Risk of Bias: unclear   - Selection process unclear - Retrospective - Avoided inappropriate exclusion   Concerns about applicability: unclear   - Limited to trauma population | Risk of Bias: high   - The index test results were not interpreted without knowledge of the reference standard results   Concerns about applicability: unclear   - The manner in which the index test was conducted and performed was not specified - Index test interpretation clear | Risk of Bias: high   - The reference standard results were not interpreted without knowledge of the index test results - Adequate descriptions of reference standards   Concerns about applicability: low   - Relevant outcomes | Risk of Bias: unclear   - The interval between the index test and the reference standard was not clearly defined - All patients received same reference standards - All patient’s outcome assessed - Timing of index tests clear unclear |
| Jaramillo, 2020 | Risk of Bias: unclear   - Selection process unclear - Retrospective - Avoided inappropriate exclusion   Concerns about applicability: unclear   - Limited to medical population | Risk of Bias: unclear   - It is unclear whether the index test results were interpreted independently of the reference standard results   Concerns about applicability: unclear   - The manner in which the index test was conducted and performed was not specified - Index test interpretation clear | Risk of Bias: unclear   - It is unclear whether the reference standard results were interpreted without knowledge of the index test results - Adequate descriptions of reference standards   Concerns about applicability: low   - Relevant outcomes | Risk of Bias: unclear   - The interval between the index test and the reference standard was not clearly defined - All patients received same reference standards - All patient’s outcome assessed - Timing of index tests clear unclear |
| Kim, 2016 | Risk of Bias: high   - Selection process non-consecutive - Prospective - Avoided inappropriate exclusion   Concerns about applicability: unclear   - Limited to medical population | Risk of Bias: high   - The index test results were not interpreted without knowledge of the reference standard results   Concerns about applicability: low   - Index test conduct and execution clear - Index test interpretation clear | Risk of Bias: high   - The reference standard results were not interpreted without knowledge of the index test results - Adequate descriptions of reference standards   Concerns about applicability: low   - Relevant outcomes | Risk of Bias: low   - Appropriate interval between index test and reference standard - All patients received same reference standards - All patient’s outcome assessed - Timing of index tests clear |
| Masoumi, 2021 | Risk of Bias: unclear   - Selection process unclear - Prospective - Avoided inappropriate exclusion   Concerns about applicability: unclear   - Limited to medical population | Risk of Bias: high   - The index test results were not interpreted without knowledge of the reference standard results   Concerns about applicability: low   - Index test conduct and execution clear - Index test interpretation clear | Risk of Bias: high   - The reference standard results were not interpreted without knowledge of the index test results - Adequate descriptions of reference standards   Concerns about applicability: low   - Relevant outcomes | Risk of Bias: unclear   - The interval between the index test and the reference standard was not clearly defined - All patients received same reference standards - All patient’s outcome assessed - Timing of index tests clear |
| Salen, 2001 | Risk of Bias: High   - Non-consecutive patients - Prospective - No clear exclusion criteria   Concerns about applicability: unclear   - Limited to medical population | Risk of Bias: high   - The index test results were not interpreted without knowledge of the reference standard results   Concerns about applicability: low   - Index test conduct and execution clear - Index test interpretation clear | Risk of Bias: unclear   - It is unclear whether the reference standard results were interpreted without knowledge of the index test results - Adequate descriptions of reference standards   Concerns about applicability: low   - Relevant outcome | Risk of Bias: unclear   - The interval between the index test and the reference standard was not clearly defined - All patients received same reference standards - All patient’s outcome assessed - Timing of index tests clear |
| Salen, 2005 | Risk of Bias: high   - Convenience sample selection - Prospective - No clear exclusion criteria     Concerns about applicability: unclear   - Limited to medical population | Risk of Bias: high   - The index test results were not interpreted without knowledge of the reference standard results   Concerns about applicability: low   - Index test conduct and execution clear - Index test interpretation clear | Risk of Bias: unclear   - It is unclear whether the reference standard results were interpreted without knowledge of the index test results - Adequate descriptions of reference standards   Concerns about applicability: low   - Relevant outcomes | Risk of Bias: high   - There was not an appropriate interval between the index test and the reference standard - All patients received same reference standards - All patient’s outcome assessed - Timing of index tests clear |
| Schuster, 2009 | Risk of Bias: high   - Convenience sample selection - Prospective and retrospective - No clear exclusion criteria     Concerns about applicability: unclear   - Limited to trauma population | Risk of Bias: low   - Index tests interpreted without knowledge of outcome   Concerns about applicability: low   - Index test conduct and interpretation clear - Index test interpretation clear | Risk of Bias: unclear   - It is unclear whether the reference standard results were interpreted without knowledge of the index test results - Adequate descriptions of reference standards   Concerns about applicability: low   - Relevant outcomes | Risk of Bias: unclear   - The interval between the index test and the reference standard was not clearly defined - All patients received same reference standards - All patient’s outcome assessed - Timing of index tests clear unclear |
| Tomruk, 2012 | Risk of Bias: low   - Selection process clear (Consecutive) - Prospective - Avoided inappropriate exclusion   Concerns about applicability: low   - Appropriate study population | Risk of Bias: unclear   - It is unclear whether the index test results were interpreted independently of the reference standard results   Concerns about applicability: low   - Index test conduct and execution clear - Index test interpretation clear | Risk of Bias: unclear   - It is unclear whether the reference standard results were interpreted without knowledge of the index test results - Adequate descriptions of reference standards   Concerns about applicability: low   - Relevant outcomes | Risk of Bias: unclear   - The interval between the index test and the reference standard was not clearly defined - All patients received same reference standards - All patient’s outcome assessed - Timing of index tests clear |

# Appendix table 4. Sensitivity analyses of POCUS using leave-one-out method

| Author, year | AUC (95% CI) | Delta AUC |
| --- | --- | --- |
| ROSC as reference standard |  |  |
| Overall studies | 0.79 (0.76-0.83) | - |
| Excluding Beckett et al. 2019 | 0.79 (0.75-0.82) | 0 |
| Excluding Chardoli et al. 2012 | 0.79 (0.76-0.83) | 0 |
| Excluding Flato et al. 2015 | 0.80 (0.76-0.83) | 0.01 |
| Excluding Gaspari et al. 2016 | 0.82 (0.78-0.85) | 0.02 |
| Excluding Jaramillo et al. 2020 | 0.80 (0.76-0.83) | 0.01 |
| Excluding Kim et al. 2016 | 0.79 (0.75-0.83) | 0 |
| Excluding Masoumi et al. 2021 | 0.81 (0.77-0.84) | 0.01 |
| Excluding Salen et al. 2005 | 0.73 (0.69-0.76) | -0.06 |
| Excluding Schuster et al. 2009 | 0.78 (0.74-0.82) | -0.01 |
| Excluding Tomruk et al. 2012 | 0.83 (0.79-0.86) | 0.01 |
| SHA as reference standard |  |  |
| Overall studies | 0.89 (0.86-0.92) | - |
| Excluding Aichinger et al. 2012 | 0.89 (0.86-0.92) | 0 |
| Excluding Beckett et al. 2019 | 0.91 (0.88-0.93) | 0.02 |
| Excluding Blaivas et al. 2001 | 0.89 (0.86-0.91) | 0 |
| Excluding Breitkreutz et al. 2010 | 0.90 (0.87-0.93) | 0.01 |
| Excluding Cebicci et al. 2014 | 0.89 (0.86-0.92) | 0 |
| Excluding Chua et al. 2017 | 0.90 (0.87-0.92) | 0.01 |
| Excluding Cureton et al. 2012 | 0.89 (0.86-0.92) | 0 |
| Excluding Gaspari et al. 2016 | 0.90 (0.87-0.92) | 0.01 |
| Excluding Israr et al. 2019 | 0.90 (0.87-0.92) | 0.01 |
| Excluding Salen et al. 2001 | 0.90 (0.87-0.92) | 0.01 |
| Excluding Salen et al. 2005 | 0.88 (0.85-0.90) | -0.01 |
| Excluding Schuster et al. 2009 | 0.90 (0.87-0.92) | 0.01 |
| SHD as reference standard |  |  |
| Overall studies | 0.74 (0.57-0.78) | - |
| Excluding Beckett et al. 2019 | 0.73 (0.57-0.78) | -0.01 |
| Excluding Cureton et al. 2012 | 0.74 (0.59-0.79) | 0 |
| Excluding Flato et al. 2015 | 0.73 (0.55-0.77) | -0.01 |
| Excluding Gaspari et al. 2016 | 0.73 (0.59-0.76) | -0.01 |
| Excluding Israr et al. 2019 | 0.75 (0.57-0.80) | 0.01 |
| Excluding Jaramillo et al. 2020 | 0.72 (0.55-0.78) | -0.02 |
| Excluding Masoumi et al. 2021 | 0.74 (0.58-0.79) | 0 |
| Excluding Salen et al. 2005 | 0.73 (0.55-0.76) | -0.01 |
| Excluding Schuster et al. 2009 | 0.75 (0.61-0.80) | 0.01 |

POCUS: point-of-care ultrasound; ROSC: return of spontaneous circulation; SHA: survival to admission; SHD: survival to discharge; AUC: Area under the curve

# Appendix Table 5. GRADE evidence profile to determine the prognostic accuracy of POCUS for ROSC, SHA, and SHD

| **ROSC** | Sensitivity 0.86 (95% CI, 0.67-0.95); Specificity 0.64 (95% CI, 0.51-0.75) | | | | | | | | | | | |
| --- | --- | --- | --- | --- | --- | --- | --- | --- | --- | --- | --- | --- |
| Outcome | Number of studies  (patients) | Study design | Factors that my decrease certainty of evidence | | | | |  | Effect per 1,000 patients tested | | | Test accuracy CoE |
|  |  |  | Risk of bias | Indirectness | Inconsistency | Imprecision | Publication bias |  | Pretest probability of 30% | Pretest probability of 40% | Pretest probability of 50% |  |
| True positive  False negative | 10  (792) | Observational | Serious^a^ | Not serious | Serious^b^ | Not serious | Serious |  | 258  (201,285)  42  (15,99) | 344 (268,380)  56  (20,132) | 430 (335,475)  70 (25,165) | ⊕◯◯◯  Very low |
| True negative  False positive | 10  (792) | Observational | Serious^a^ | Not serious | Serious^b^ | Not serious | Serious |  | 448 (357,525)  252  (175,343) | 384 (306,450)  216  (150,294) | 320 (255,375)  180  (125,245) | ⊕◯◯◯  Very low |
| **SHA** | Sensitivity 0.89 (95% CI, 0.80-0.94); Specificity 0.73 (95% CI, 0.63-0.81) | | | | | | | | | | | |
| Outcome | Number of studies  (patients) | Study design | Factors that my decrease certainty of evidence | | | | |  | Effect per 1,000 patients tested | | | Test accuracy CoE |
|  |  |  | Risk of bias | Indirectness | Inconsistency | Imprecision | Publication bias |  | Pretest probability of 10% | Pretest probability of 20% | Pretest probability of 30% |  |
| True positive  False negative | 12  (930) | Observational | Serious^a^ | Not serious | Not serious^c^ | Not serious | Not serious |  | 89  (80,94) 11  (6,20) | 178 (160,188) 22  (12,40) | 267 (240,282) 33  (18,60) | ⊕⊕⊕◯  Moderate |
| True negative  False positive | 12  (930) | Observational | Serious^a^ | Not serious | Serious^c^ | Not serious | Not serious |  | 657 (567,729) 243 (171,333) | 584 (504,648) 216 (152,296) | 511 (441,567) 189 (133,259) | ⊕⊕◯◯  Low |
| **SHD** | Sensitivity 0.79 (95% CI, 0.58-0.91); Specificity 0.58 (95% CI, 0.47-0.68) | | | | | | | | | | | |
| Outcome | Number of studies  (patients) | Study design | Factors that my decrease certainty of evidence | | | | |  | Effect per 1,000 patients tested | | | Test accuracy CoE |
|  |  |  | Risk of bias | Indirectness | Inconsistency | Imprecision | Publication bias |  | Pretest probability of 1% | Pretest probability of 3% | Pretest probability of 10% |  |
| True positive  False negative | 9  (820) | Observational | Serious^a^ | Not serious | Not serious^d^ | Not serious | Not serious |  | 8  (6,9)  2  (1,4) | 24  (17,27) 6  (3,13) | 79  (58,91) 21  (9,42) | ⊕⊕⊕◯  Moderate |
| True negative  False positive | 9  (820) | Observational | Serious^a^ | Not serious | Serious^d^ | Serious | Not serious |  | 574 (465,673) 416 (317,525) | 563 (456,660) 407 (310,514) | 522 (423,612) 378 (288,477) | ⊕◯◯◯  Very low |

^a^ As assessed by QUADAS-2, seven studies (39%) were rated as having a high risk of bias in patient selection, primarily due to the use of convenience or non-random sampling. In most studies (83%), unclear or high risks of bias were identified in the index test and reference standard, mainly due to the absence of pre-defined criteria for cardiac activity and a lack of a clear POCUS protocol. In the flow and timing domain, the absence of blinding in most studies (89%) raised concerns about the potential for decreased resuscitation efforts and self-fulfilling prophecies. We downgraded one level.

^b^ For individual studies, sensitivity estimates ranged from 0.43 to 1, and specificity estimates ranged from 0.33 to 0.87. We downgraded the sensitivity and specificity by one level.

^c^ For individual studies, sensitivity estimates ranged from 0.63 to 1, and specificity estimates ranged from 0.41 to 0.90. We downgraded the specificity by one level.

^d^ For individual studies, sensitivity estimates ranged from 0.83 to 1, and specificity estimates ranged from 0.19 to 0.76. We downgraded the specificity by one level.

The pretest probability was approximately the first interquartile, median, and third interquartile prevalence values from the included studies.

POCUS: point-of-care ultrasound; ROSC: return of spontaneous circulation; SHA: survival to admission; SHD: survival to discharge; CoE: certainty of evidence; QUADAS-2: Quality Assessment of Diagnostic Accuracy Studies-2

GRADE Certainty of the evidence

High: we are very confident that the true effect lies close to that of the estimate of the effect. Moderate: we are moderately confident in the effect estimate: the true effect is likely to be close to the estimate of the effect, but there is a possibility that it is substantially different. Low: our confidence in the effect estimate is limited: the true effect may be substantially different from the estimate of the effect. Very low: we have very little confidence in the effect estimate: the true effect is likely to be substantially different from the estimate of effect
